# Evaluation of the Effect of Oral Pyridostigmine on the Ileus after Abdominal Surgery: A Blinded Randomized Clinical Trial

**DOI:** 10.3390/jcm7050104

**Published:** 2018-05-06

**Authors:** Abdulbaset Maleknejad, Alireza Khazaei, Salehoddin Bouya

**Affiliations:** 1 Department of General Surgery, Medicine School, Zahedan University of Medical Sciences, Zahedan 9816743463, Iran; ravan_speak@yahoo.com; 2 Department of Nephrology and internal medicine, Zahedan University of Medical Sciences, Zahedan 9816743463, Iran; hresearchh@gmail.com

**Keywords:** oral pyridostigmine, postoperative ileus, pain management

## Abstract

Postoperative ileus is one of the most important and common complications after abdominal surgery. This single-blind randomized clinical trial study was conducted with the aim of evaluating the effect of oral pyridostigmine (60 mg) on the duration and frequency of response to the treatment of ileus after abdominal surgery on 40 patients in two 20-subject groups of oral pyridostigmine (interventional) and starch (control) in 2015. All 40 people completed the study process and entered the final analysis. In the oral Pyridostigmin group (60 mg) the mean response time for the disposal of gas and stool were 5.4 ± 4.7 h and 4.9 ± 3.4 h, respectively. Most of the participants 10 (50%) (Disposal of stool) responded to treatment 4 h after the administration of oral pyridostigmine. In the starch group, the mean response time for the disposal of gas and stool were 32.4 ± 9.9 h and 36.2 ± 10.3 h, respectively. The mean treatment response time in two groups showed a significant difference between both groups (*p* = 0.001). Regarding the frequency of response to treatment (disposal of gas or stool) in the 24-h period after the initiation of treatment in the oral pyridostigmine group, 95% (*n* = 19) of the subjects responded to the treatment in the first 24 h. However, in the starch group, only 50% (*n* = 10) responded to treatment in the first 24 h, the results showed a significant difference between the two groups (*p* = 0.001). The results indicate that oral pyridostigmine can be used as a simple and effective treatment for gastrointestinal ileus.

## 1. Introduction

Postoperative ileus (POI) is one of the most important and common complications experienced after abdominal surgeries and it costs 1.5 billion US $ a year in the United States [1,2]. Generally, POI is known as a disorder of the gastrointestinal tract after abdominal surgery and it is capable of affecting the entire digestive system whose time incidence for each section of this system is different [3,4,5]. After surgery, the movements of the small intestine start sooner than the rest of the digestive tract. Most times, it occurs in the first few hours after surgery, while gastric motility returns in 1–2 days and colon motility returns in 2–3 days [6]. In the colon, first the movements of the primary and then the end parts are established [3]. There are several factors that contribute to the development of the ileus, and they have been discussed in detail. Studies have shown that this is caused by an unplanned and irregular gastrointestinal activity [7]. Stomach emptying due to increased pyloric tone is blocked [3]. The movements of the small and large intestines are slow and even reversed [4]. Its pathophysiology is not fully understood, but the factors involved include the following: sympathetic local and spinal reflexes as well as inflammatory mediators [8]. Various studies have shown that the intestinal manipulations during a surgical situation affect intestinal walls and lead to inflammatory response, thereby resulting in the introduction of fluids and germs into the intestine, and the activation of macrophages result in the liberation of kinetically active substances, nitric oxide (NO) and prostaglandins, resulting in the release of large amounts of inflammatory mediators and the secretion of proinflammatory cytokines such as interleukin-1b, interleukin-6 and monocyte chemotactic protein-1, resulting in the decreased movements of the digestive system and the ileus [9,10,11]. The critical role of the proinflammatory cytokines, NO and prostaglandins in causing POI has been confirmed through the use of pharmacological and genetic methods [12,13]. The role of the nervous system has also been explained as the secretion of a vasoactive intestinal peptide (VIP), substance P (SP) that has an inhibitory effect on intestinal movements [14,15]. Although postoperative ileus has no definitive cure, there are a number of different approaches for its prevention. These include the correction of potassium deficiency, early mobilization of the patient, the use of a stomach tube, and laparoscopic surgery, which results in less ileus due to less manipulation of the intestines. The most commonly used treatment for ileus pain is the administration of opioids, which, although it can improve the pain relief, due to the effects on the intestine, it slows down intestinal movement in the long run and causes ileus exacerbation. On the other hand, other treatments cannot be used especially in patients with impaired function or a low level of consciousness [3,14,16,17,18]. Medical treatments of the ileus include the administration of metoclopramide, Cisapride and erythromycin, which reduce the risk of ileus, as a result of the cholinergic effects [19,20,21]. In addition, cholinergic drugs such as neostigmine are administered in the intravenous form [15,22]. Although intravenous neostigmine is a common treatment, it requires closely observing patients while administering neostigmine for cardiac side effects, and, compared to the ease of using oral pyridostigmine, the use of oral pyridostigmine is better due to the better availability and affordability and the easier way to use it for effective results. Thus, it can be considered a better treatment to control the pain caused by ileus, which is regarded as an important clinical complication after surgery [23]. Ileus can increase a number of embolisms, postoperative pain, hospitalization days, and, consequently, an increase in the costs of the health system. The aim of this study was to evaluate the effect of oral pyridostigmine on the duration and frequency of response to treatment of ileus after abdominal surgery.

## 2. Materials and Methods

### 2.1. Study Design and Registration 

This single blind (patients) clinical trial was conducted from January to May 2015, in three general surgery units at an educational hospital in Zahedan, Iran. This study was approved by the Ethics Committee of Zahedan University of Medical Sciences and the Ethics Committee of the place where research was conducted (Ethic code: IR.ZAUMS.REC.1393.966). The clinical trial was approved by the Iranian Registry of Clinical Trials (IRCT) under No: IRCT20180123038484N1. The CONSORT checklist was used to report the study [24].

### 2.2. Participants

All participants were evaluated for the inclusion criteria, which entailed having ileus for three or more than three days [25,26] and consenting to participation in the study. The exclusion criteria were as follows: heart rate of less than 60 times per minute, blood pressure of less than 90 mmHg, age below 18 years, finding mechanical causes for inactivity of the intestines, heart problems, colon cancer and small bowel cancer. Initially, after the abdominal surgery and one day after stabilization of their condition, the patients were called to assemble in one place and were asked to express their tendency to participate in the study. The researcher fully explained the study process to the participants and their family. Being assured of their participation in the study, written consent was obtained from them.

### 2.3. Intervention

**Figure 1:**
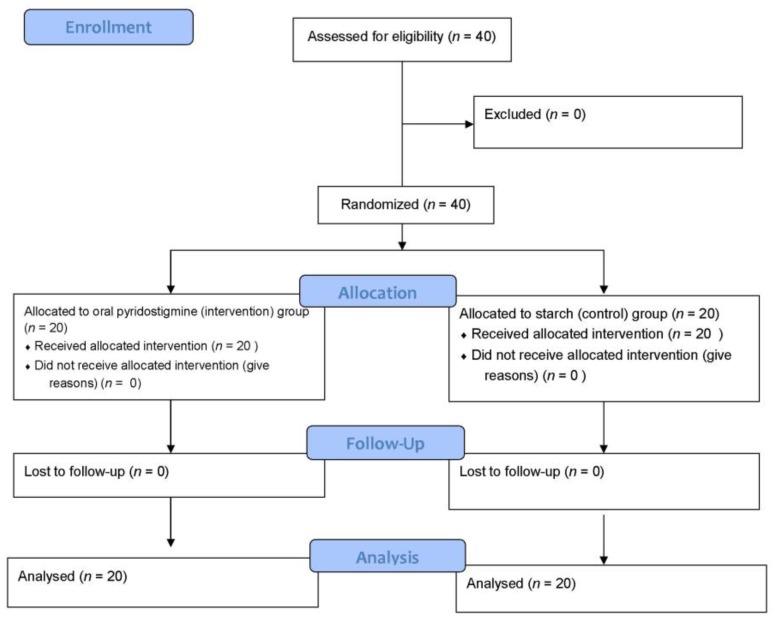
The study flow diagram.

This study was based on a similar study that suggested that at least 20 subjects were required in each group to determine the difference between the two groups (95% confidence interval and 5% error rate) [27]. The patients were examined for the inclusion criteria and 40 patients were randomly selected and assigned to four blocks of 10 patients each by a randomized block sampling method. Using a factorial design, patients were randomly assigned into each group based on type of operation and inclusion criteria. This study was single blind, patients were aware of being involved in the study, but not aware of which group they are in at the time of intervention. The patients were divided into intervention and control groups. In the intervention group, the patients received 60 mg of oral pyridostigmine twice a day via NGTUBE, which started after three days of ileus [23]. In the control group, the patients received starch via NGTUBE twice a day, which started after three days of ileus. The treatment response time and the treatment response frequency were measured by observing or questioning the patient for gas and fecal excretion, which served as the main criteria for measuring the effect of the drug (Figure 1).

### 2.4. Data Collection and Analysis

The collected data included the demographic characteristics of the participants (gender, age, kind of surgery and the time of the surgical procedure) and therapy groups (intervention (oral pyridostigmine) and control group (starch)) and treatment response frequency. To describe the demographic characteristics, descriptive tests (percentage, frequency and mean) were used. An independent *t*-test was used to compare the mean treatment response time between the two groups considering the normality of the data. A chi-square test was used for comparison of two groups. An independent *t*-test was also used to compare the treatment response frequency between the two groups considering the normality of the data.

## 3. Results

This section may be divided by subheadings. It should provide a concise and precise description of the experimental results, their interpretation as well as the experimental conclusions that can be drawn.

### 3.1. Participants

A total of 40 people were selected for this study and all of them were present until the final stage of the study. The demographic characteristics in both the pyridostigmine and starch groups were similar. In the oral pyridostigmine group, the mean age of the participants was 60.5 ± 2.4 years, and, in the starch group, it was 61.9 ± 1.7 years, and the mean age in both groups was 60.9 ± 2.1 years. Gender in the pyridostigmine group was equal in the number of males and females and in the starch group. Most of the participants were male *n* = 12 (60%), in two groups more patients had a cesarean surgery, and the mean time of the surgical procedure was 2.2 h (Table 1).

### 3.2. Main Results

#### Mean Response Time

Regarding the first objective, in the Oral Pyridostigmin group, the mean response times for the disposal of gas and stool were 5.4 ± 4.7 h and 4.9 ± 3.4 h, respectively. Most of the participants 10 (50%) (disposal of stool) responded to treatment 4 h after the administration of oral pyridostigmine. In the starch group, the mean response times for the disposal of gas and stool were 32.4 ± 9.9 h and 36.2 ± 10.3 h, respectively. The results of *t*-test showed that there was a significant difference between the two groups and patients in the Oral Pyridostigmine group responded to treatment with much less time (*p* = 0.001) (Table 2).

Concerning the second objective, the frequency of treatment response (disposal of gas or stool) in the first 24 h after the commencement of treatment in the oral pyridostigmine group, 95% (*n* = 19) of the subjects had treatment response in the first 24 h. However, in the starch group, only 50% (*n* = 10) responded to treatment in the first 24 h such that the chi-square test showed a significant difference between the two groups (*p* = 0.001) (Table 3). In addition, the results showed that the success treatment rate is better in patients with cesarean (*n* = 7) delivery than patients having appendectomy and cholecystectomy (intestinal operation) (*n* = 3). No side effects were observed in the two study groups.

## 4. Discussion

The aim of this study was to investigate the effect of oral pyridostigmine on the time and frequency of treatment response to ileus after abdominal surgery on 40 patients in both oral pyridostigmine and starch groups. The results of this study showed that patients taking oral pyridostigmine responded much better. There was no similar study that performed exactly the same intervention. In Bharucha’s study, which was conducted on 10 patients diagnosed with autonomic neuropathy who received 60 mg oral pyridostigmine three times a day (TID) for six weeks, the results revealed that oral pyridostigmine improves the symptoms of gastro intestinal (GI) (daily and weekly bowel diaries, colonic motility, gastroduodenal motility) and Scintigraphic transit: gastric emptying, small bowel, and colonic transit [28]. In addition, Soufi-Afshar’s study on 68 adults with constipation who used Pyridostigmine 60 mg TID and bisacodyl for four weeks showed that there was a similar significant improvement (pre vs. post-therapy) in both groups (bisacodyl and pyridostigmine) in regard to Bristol stool score, frequency of bowel movements, and straining [29]. In addition, different case reports [30,31] and case series [23] studies have shown that oral pyridostigmine was effective and safe in all cases. However, in the case of intravenous pyridostigmine in rats, the study conducted by Emirleroglu et al. in 2011 showed that the early onset of post-operative neostigmine nutrition is associated with decreased postoperative ileus and an improvement in colonic anastomosis [32]. The study conducted by Oigaard et al. in 1975 showed a significant increase in AD electrical and motor activity postlaparotomy [33]. In addition, in a study by Fanaie et al. in 2007, neostigmine was shown to have a positive therapeutic effect on the reduction of ileus after abdominal surgery [27]. In most patients with ileus, decision-making about the type of treatment in different people is based on different patient conditions and includes medical treatment, protective treatment, colonoscopy and surgery [34]. Colonoscopies are successful in 70% of patients [35]. However, a colonoscopy was difficult in these patients and the morbidity and mortality rates were 3 and 1%, respectively. The surgical intervention has many complications and should be performed in patients with ischemia, perforation, or failure in colonoscopy [27]. Consequently, due to the complications and success rate mentioned for other methods in comparison with pyridostigmine treatment, this method is associated with very few complications, and, most importantly, it shortens the duration of hospitalization. However, it is noteworthy that treatment with parasmpatomythmic drugs such as pyridostigmine and neostigmine is not risk-free; particularly, there is a risk of developing severe bradycardia in patients with bradyarrhythmias or beta-adrenergic antagonists. Due to limitation in access to different types of participants, more participants in the present study were from the caesarean ward. According to the individual studies, POI is a major problem in caesarean wards due to abdominal pain and distention, oral intake difficulty, breast feeding disability, and prolonged hospital stay like that other intestinal surgeries [36,37,38]. 

The strength of this study is in the performance of clinical trials. The limitation of this study is that a long time is required to select and enter the patients into the study and also the sample size of the study.

## 5. Conclusions

The overall results of the study indicate a positive effect of oral pyridostigmine as a simple and effective treatment of the ileus colon; however, because of the low sample size and low studies conducted in this field, it has been suggested that studies should be conducted with a larger volume and more time is required to better assess the therapeutic effect of this drug.

## Figures and Tables

**Table 1:** Demographic characteristics of participants in two groups.

Variables		Oral Pyridostigmine Group Mean ± SD	Starch Group Mean ± SD
Age		60.5 ± 2.4	61.9± 1.7
Gender	Male	*n* (%) *n* = 20	*n* (%) *n* = 20
		10(50%)	12(60%)
	Female	10(50%)	8(40%)
Kind of surgery	Caesarean	10(50%)	10(50%)
	Cholecystectomy	5(25%)	5(25%)
	Antrectomy	3(15%)	3(15%)
	Appendectomy	2(10%)	2(10%)
Time of the surgical procedure (h)		2.1 ± 1.2	2.4 ± 1.4

**Table 2:** Comparison of the effects of oral pyridostigmin and starch on response time in patients with abdominal ileus.

Variable	The Time to Start Responding to Treatment	Oral Pyridostigmine Group		The Time to Start Responding to Treatment	Starch Group (Control)		*p*-Value
		Disposal of Gas *n* (%)	Disposal of Stool *n* (%)		Disposal of Gas *n* (%)	Disposal of Stool *n* (%)	
Treatment response time	2 h	4(20%)	5(25%)	12 h	4(20%)	3(15%)	*t*-test
	4 h	9(45%)	10(50%)	18 h	6(30%)	7(35%)	
	6 h	4(20%)	3(15%)	24 h	4(20%)	3(15%)	
	8 h	2(10%)	2(10%)	30 h	2(10%)	3(15%)	
	24 h	1(5%)	0	36 h	3(15%)	2(10%)	
				48 h	1(5%)	2(10%)	
Mean treatment response time	-	5.4 ± 4.7	4.9 ± 3.4	-	32.4 ± 9.9	36.2 ± 10.3	0.001

**Table 3:** Comparison of the effect of oral pyridostigmin and starch on the frequency of response to the treatment of abdominal ileus.

Variable	The Time to Start Responding to Treatment	Oral Pyridostigmine Group	The Time to Start Responding to Treatment	Starch Group (Control)	*p*-Value
		*n* (%)		*n* (%)	
Treatment response time	2 h	4(20%)	12 h	4(20%)	chi-square test
	4 h	9(45%)	18 h	6(30%)	
	6 h	4(20%)	24 h	4(20%)	
	8 h	2(10%)	30 h	2(10%)	
	24 h	1(5%)	36 h	3(15%)	
			48 h	1(5%)	
Mean treatment response time	-	5.4 ± 4.7	-	32.4 ± 9.9	0.001
